# *De novo* transcriptome analysis using 454 pyrosequencing of the Himalayan Mayapple, *Podophyllum hexandrum*

**DOI:** 10.1186/1471-2164-14-748

**Published:** 2013-11-01

**Authors:** Dipto Bhattacharyya, Ragini Sinha, Saptarshi Hazra, Riddhi Datta, Sharmila Chattopadhyay

**Affiliations:** 1 Plant Biology Laboratory, Drug Development/Diagnostics & Biotechnology Division, CSIR-Indian Institute Chemical Biology, 4 Raja S. C. Mullick Road, Kolkata 700032, India; 2 Current address: Division of Biotechnology, Chonbuk National University, 79 Gobong-ro, Iksan-si, Jeollabuk-do 570-752, Republic of Korea

**Keywords:** 454 pyrosequencing, *Podophyllum hexandrum*, Podophyllotoxin biosynthesis, Transcriptome

## Abstract

**Background:**

The Himalayan or Indian Mayapple (*Podophyllum hexandrum* Royle) produces podophyllotoxin, which is used in the production of semisynthetic anticancer drugs. High throughput transcriptome sequences or genomic sequence data from the Indian Mayapple are essential for further understanding of the podophyllotoxin biosynthetic pathway.

**Results:**

454 pyrosequencing of a *P. hexandrum* cell culture normalized cDNA library generated 2,667,207 raw reads and 1,503,232 high quality reads, with an average read length of 138 bp. The denovo assembly was performed by Newbler using default and optimized parameters. The optimized parameter generated 40, 380 assembled sequences, comprising 12,940 contigs and 27,440 singlets which resulted in better assembly as compared to default parameters. BLASTX analysis resulted in the annotation of 40,380 contigs/singlet using a cut-off value of ≤1E-03. High similarity to *Medicago truncatula* using optimized parameters and to *Populus trichocarpa* using default parameters was noted. The Kyoto encyclopedia of genes and genomes (KEGG) analysis using KEGG Automatic Annotation Server (KAAS) combined with domain analysis of the assembled transcripts revealed putative members of secondary metabolism pathways that may be involved in podophyllotoxin biosynthesis. A proposed schematic pathway for phenylpropanoids and podophyllotoxin biosynthesis was generated. Expression profiling was carried out based on fragments per kilobase of exon per million fragments (FPKM). 1036 simple sequence repeats were predicted in the *P. hexandrum* sequences. Sixty-nine transcripts were mapped to 99 mature and precursor microRNAs from the plant microRNA database. Around 961 transcripts containing transcription factor domains were noted. High performance liquid chromatography analysis showed the peak accumulation of podophyllotoxin in 12-day cell suspension cultures. A comparative qRT-PCR analysis of phenylpropanoid pathway genes identified in the present data was performed to analyze their expression patterns in 12-day cell culture, callus and rhizome.

**Conclusions:**

The present data will help the identification of the potential genes and transcription factors involved in podophyllotoxin biosynthesis in *P. hexandrum*. The assembled transcripts could serve as potential candidates for marker discovery and conservation, which should form the foundations for future endeavors.

## Background

*Podophyllum hexandrum* Royle, commonly referred to as the Himalayan/Indian Mayapple, is an endangered perennial herb belonging to the family Berberidaceae, which is distributed on the lower slopes of the Himalayas in scrub and forest, from Afghanistan to central China [1]. Roots and rhizomes of *P. hexandrum* contain lignans, such as podophyllotoxin and other related aryltetralin lignans [2], which are present in *Podophyllum* spp. but are not restricted to this genus. Extracts of *Podophyllum* spp. have been used by diverse cultures as antidotes against poisons and as cathartic, purgative, anthelmintic, vesicant and suicidal agents [3]. A crude resin extract of *Podophyllum* spp., podophyllin, was included in the US Pharmacopoeia in 1820, and this resin has been prescribed to treat venereal warts. Podophyllotoxin has been used as the starting compound for the production of the semi-synthetic drugs etoposide (VP-16-213), teniposide (VM-26) and etopophos, which are used to treat lung and testicular cancers [4], leukaemia and rheumatoid arthritis [5]. A recent review [6] stated that species containing podophyllotoxin were traditionally used as folk-remedies in China, Japan, India and the United States to treat several illnesses, including gout, tuberculosis, syphilis, warts and various tumors. The Indian species, *P. Hexandrum*, contains three times more Podophyllotoxin than its American counterpart, *P. peltatum*, which contains other lignans, such as α- and β-peltatins [7,8]. However, peltatins do not contribute to the anti-cancer properties of the plant [9].

To meet the commercial demand, podophyllotoxin has been extracted from the rhizomes of *P. hexandrum* and *P. peltatum* collected in the wild. The chemical synthesis of podophyllotoxin is possible, but not economically feasible [10]. Therefore, large quantities of rhizomes have been collected indiscriminately to meet the ever-increasing demands of modern medicine. Severe habitat destruction and over-collection has created acute depletion in the population of this herb. Together with a lack of organized cultivation, this has led to *P. hexandrum* being classified as a critically endangered species in the Himalayan region [11,12].

In addition to this genus, other plants, including *Linum album*, *Juniperus chinensis* and *Callitris drummondii*, have been investigated for the *in vitro* production of podophyllotoxin and its derivatives [13]. However, the production of podophyllotoxin using cell cultures may not be sufficient for biotechnological production systems [14].

The complete sequences of dirigent protein oxidase (DPO), secoisolariciresinol dehydrogenase (SDG) and cinnamyl alcohol dehydrogenase (CAD) from *P. hexandrum* have been deposited in the National Centre for Biotechnology Information (NCBI). Lignan biosynthesis involves mechanisms for enantioselective dimerization. DPO affects the selective coupling of the coniferyl alcohol radical to produce (+)-pinoresinol [15] and pinoresinol reductase converts pinoresinol to secoisolariciresinol via lariciresinol [16]. Then, (-)-secoisolariciresinol is converted to (-)-matairesinol by SDG [17]. Matairesinol is a starting point for the biosynthesis of podophyllotoxin. One possible pathway is that matairesinol is converted to yatein and then to podophyllotoxin via deoxypodophyllotoxin [18]. Another direct pathway to podophyllotoxin from matairesinol via thujaplicatin has been proposed [19]. Although the podophyllotoxin biosynthetic pathway is reasonably well characterized and several cDNAs have been reported, the transformation from matairesinol to podophyllotoxin involves hydroxylation, methylations and methylenedioxy bridge formation, and these late steps are yet to be characterized. A recent report revealed two cytochrome P450 enzymes in *P. hexandrum* and *P. peltatum* that are capable of converting (-)-matairesinol into (-)-pluviatolide by catalyzing the formation of a methylenedioxy bridge [20].

*De novo* transcriptome analysis of next-generation sequencing data is an appropriate technique for identifying unknown genes in non-model organisms [21]. Expressed sequence tag (EST) sequencing, which excludes non-coding and repetitive DNA components, is a cost-effective and frequently used strategy to analyze the transcriptome. Here, we sequenced the transcriptome of *P. hexandrum* cell culture using the 454 GS-FLX Titanium technology, assembled the raw reads using three assemblers, and chose an assembler with the best performance. Finally, functional annotation, FPKM value, domain analysis, transcription factors (TFs) and simple sequence repeat (SSR) identification, and miRNA targeted transcript identification, were determined. Domains from the identified transcripts that could represent downstream genes encoding enzymes that catalyze the late steps in podophyllotoxin biosynthesis were also identified. The data from this study will form the basis for future studies towards the isolation and characterization of the podophyllotoxin biosynthetic pathway genes from *P. Hexandrum*.

## Results and discussion

### 454 sequencing of the Mayapple cell culture transcriptome

Clonally amplified cDNA library beads obtained from emulsion-based clonal amplification (emPCR amplification) reactions were subjected to two experimental runs on a Pico Titre Plate (PTP) for sequencing using Roche 454 GS FLX pyrosequencing chemistry. A total of 2,667,207 raw reads (Table 1) were obtained, and the low quality reads, adapters and primer sequences were removed using PRINSEQ [22]. After quality filtration and adapter trimming of raw reads, 1,503,232 high quality (HQ) reads with an average read length of 138 bp was obtained. The high quality reads were uniqued and mapped to Rfam, non coding RNA database. Approximately, 50% filtered reads were obtained and used for further analysis.

**Table 1:** Overview of sequencing reads and reads after preprocessing

**Statistics**	***P. hexandrum*** **cell culture sample 454 pyrosequencing**
Sequencing reads before preprocessing	2,667,207
Number of HQ reads	1,503,232
Average length of HQ read (bp)	138 bp
Total length (bp)	207,124,754 bp

### Comparison between default and optimized parameters of Newbler

**Figure 1:**
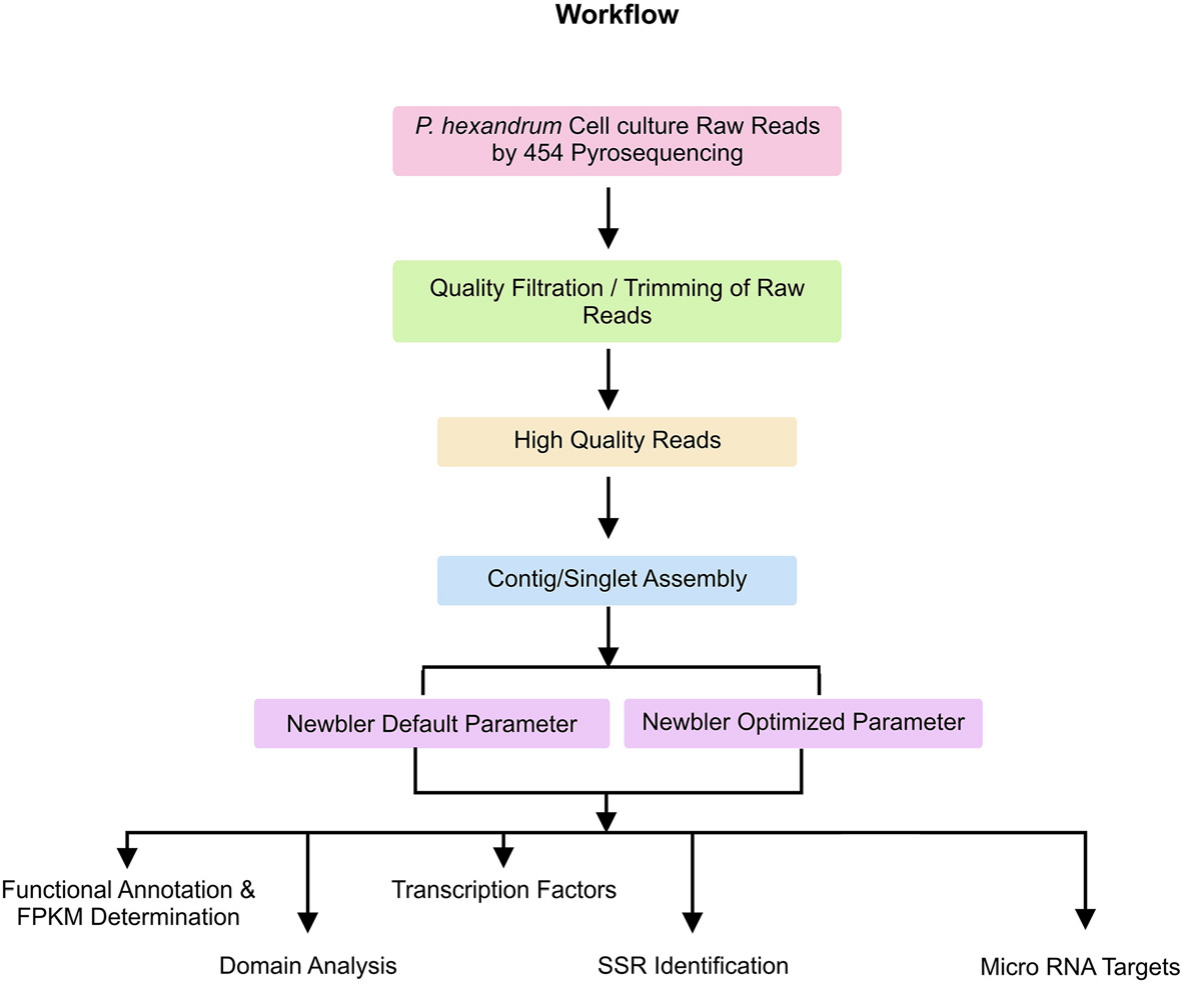
Workflow for 454 sequencing, assembly, annotation, and other analyses done.

Here we present a simple workflow for 454 sequencing, assembly, annotation and other analyses (Figure 1). Newbler is frequently used in de novo pyrosequencing projects [23]. The comparative denovo assembly was carried out using Newbler with default and optimized parameters [24]. The optimized parameter generated 40,380 assembled sequences, comprising 12,940 contigs and 27,440 singlets with an N50 of 463 and 240 for contigs and singlets respectively. Newbler with optimized parameters gave the best results in terms of the numbers of assembled contigs and singlet, N50, mean contig/singlets length and total bases of contigs /singlets (bp) (Table 2). Additional file 1 (A and B) shows the distribution of contig lengths generated by Newbler using default and optimized parameters respectively. Further analysis of the singlet generated by Newbler default assembly were excluded because their mean length was below 200 bp.

**Table 2:** Assembly results of 454 data by using Newbler default and optimized parameters

**Description**	**Newbler (Default parameter)**		**Newbler (Optimized parameter)**	
	**Contigs**	**Singlets**	**Contigs**	**Singlets**
Number of contigs/Singlets	3372	62937	12940	27440
Total bases of contigs/Singlets(bp)	1143488	12156572	5928039	7033852
Mean contig/Singlet length	339.112	193.154	458.117	256.335
Contig/Singlet N50	339	190	463	240
Max Contig/Singlet size	2167	297	4915	399

### Functional annotation of assembled transcripts and determination of FPKM values

**Figure 2:**
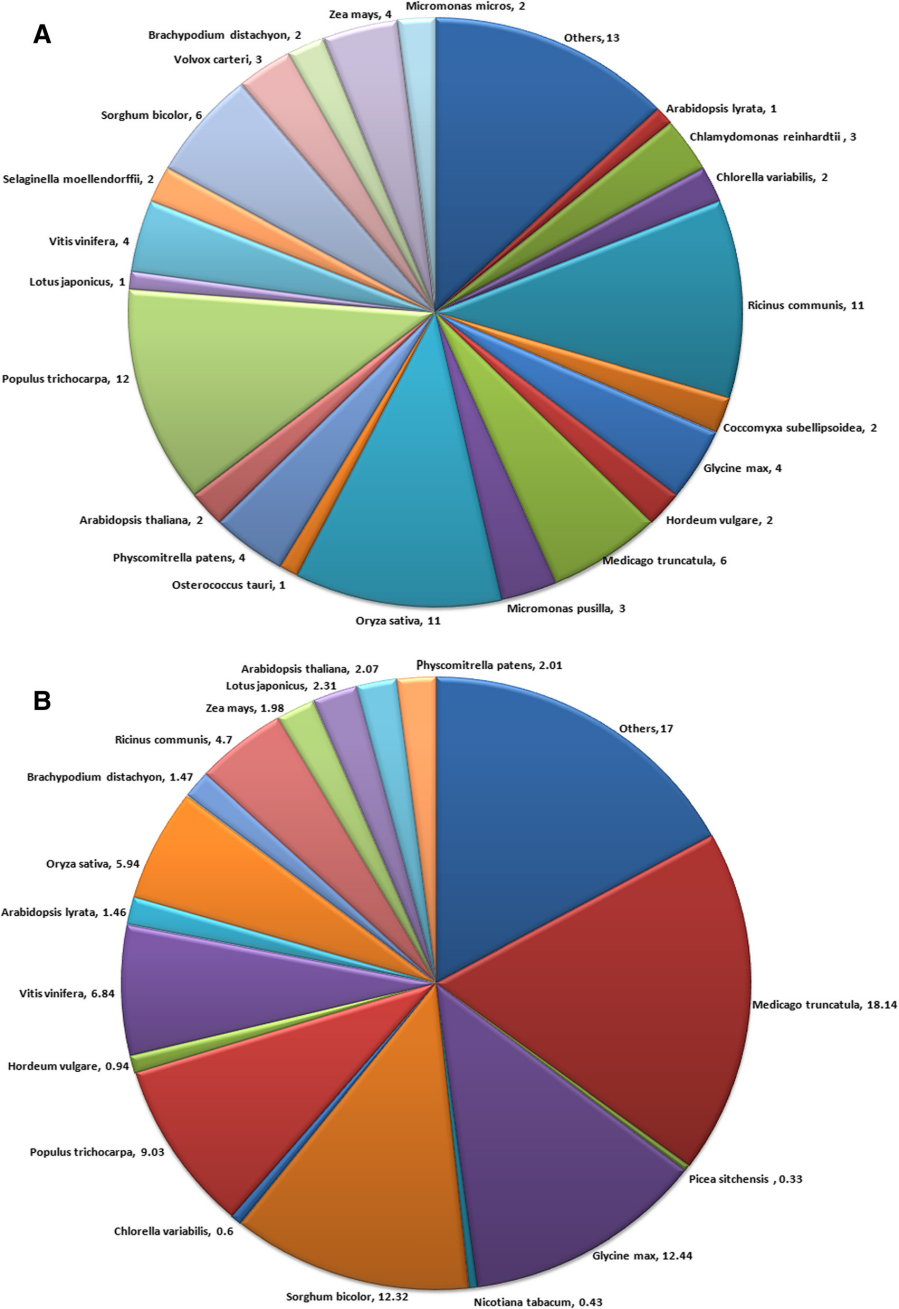
BLASTX Top-Hit species distribution of transcript contigs and singlet generated by Newbler assembly using A) default and B) optimized parameters.

**Figure 3:**
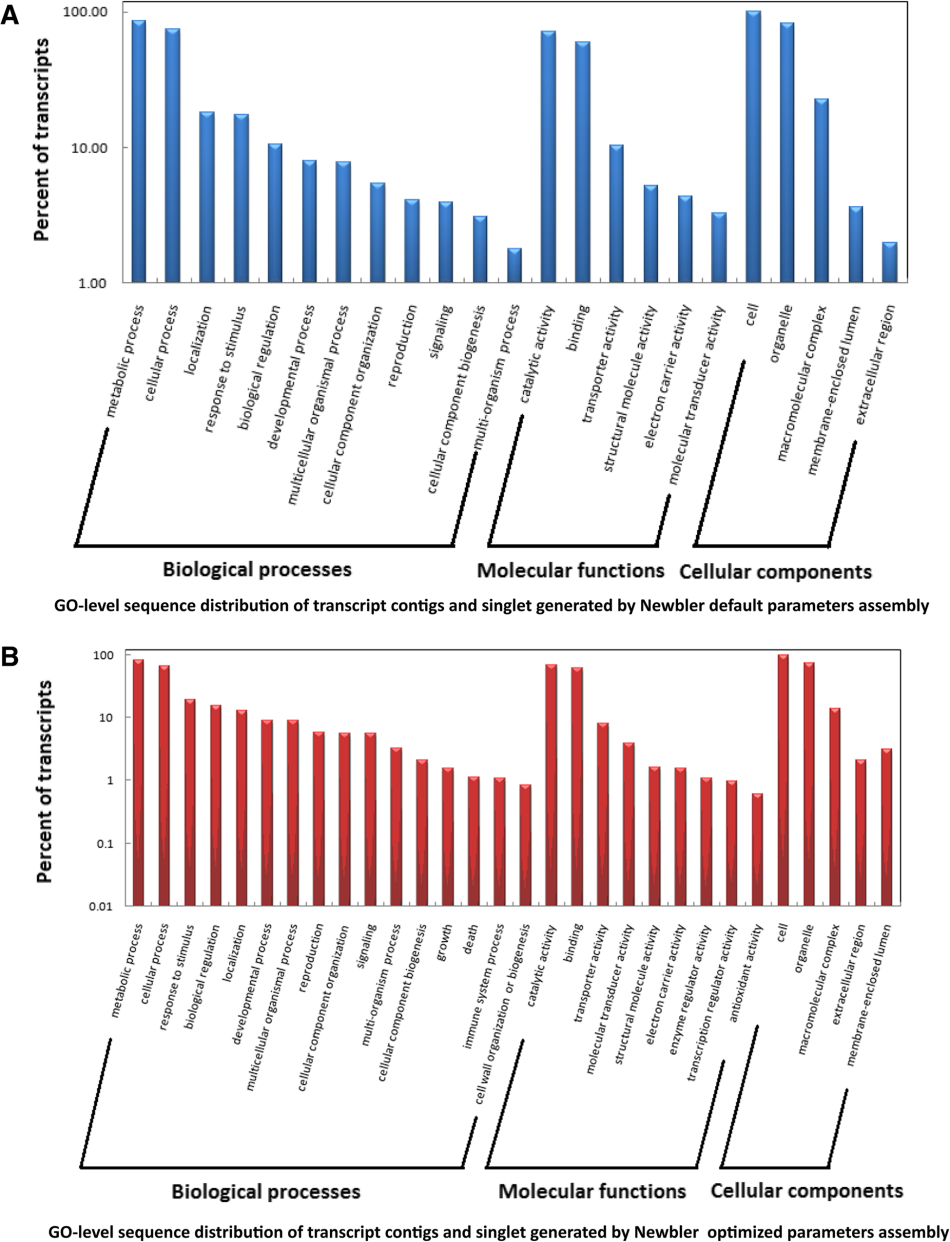
**Gene Ontology-level sequence distribution.** GO-level sequence distribution for Biological processes, molecular functions, and cellular components of transcript contigs and singlet generated by Newbler assembly using **A)** default parameters and **B)** optimized parameters.

The annotation of transcripts was carried out using green plants of non-redundant (nr) protein database at NCBI by BLASTX [25]. BLASTX resulted in the annotation of 3,249 contigs out of 3,372 assembled contigs obtained using Newbler default parameters (Additional file 2) whereas 40,380 transcripts from among 12,940 contigs and 27,440 singlet generated using Newbler optimized parameters (Additional file 3). Using default parameters, transcripts showed significant similarity with *P.trichocarpa* followed by *Oryza sativa*, *Ricinus communis* and so forth, (Figure 2A) while, using optimized parameters, significant similarity was achieved with *M. truncatula*, followed by *Glycine Max*, *Sorghum bicolor*, *P. trichocarpa* and others (Figure 2B). GO assignments [26] were used to classify the functions of the predicted transcript contigs to determine the E-value distribution, sequence similarity distribution, evidence code distribution of sequences, evidence code distribution of BLAST hits, annotation score distribution, annotation distribution and GO-level distribution of transcripts generated by default (Additional file 4) and optimized Newbler assembly ( Additional file 5). The GO annotation of transcripts from Newbler default and optimized assemblies are represented in Additional file 6 and Additional file 7 respectively. GO-level sequence distribution for Biological processes, Molecular functions and Cellular components of the transcript contigs and singlet generated by Newbler default and optimized assembly are shown in Figure 3 (A, B respectively). Digital expression profiling by FPKM of each transcript generated from Newbler using default and optimized parameters assembly were also determined (Additional file 8 and Additional file 9).

To identify the biological pathways that are active in *P. hexandrum* cell culture, we mapped the annotated sequences to the reference canonical pathways in KEGG [27]. Among the annotated sequences generated by Newbler using default and optimized parameters, 321 and 1069 unique non-redundant sequences were involved in a particular KEGG pathway of which 32 and 100 unique sequences could be assigned to secondary metabolism respectively (Additional file 10 and Additional file 11).

### Protein domains encoded by the *P. hexandrum* transcriptome that may represent genes involved in podophyllotoxin biosynthesis

We were interested in probable podophyllotoxin pathway genes that could be identified from the transcriptome, therefore Conserved Domains Database (CDD), Pfam, and Tigr databases were searched for domains encoding CADs, monooxygenases, peroxidases (POD), pinoresinol reductases, DPOs, SDGs, and methyl transferases. Our search identified transcripts coding for domains of CAD, SDG, monooxygenase, POD, methyl transferase, NADB Rossmann superfamily, Flavin utilizing monooxygenases superfamily, Uroporphyrinogen decarboxylase methyltransferase (URO-D CIMS) like superfamily, Isoprene-C2-like reductase (ISOPREN C2) like superfamily, Cytochrome oxidase (CypX) superfamily and Oxidoreductase q1 (Oxidored q1) superfamily (Additional file 12 and Additional file 13). According to the hypothetical scheme of podophyllotoxin pathway [18], matairesinol is converted to podophyllotoxin by two consecutive methyl group additions forming a compound like yatein. We were also interested in finding methyl transferases that can transfer two methyl groups to the same substrate at the same time. A Uroporphyrinogen IIIC methyl transferase from *P. hexandrum* was identified in our previous studies, which is known to function in transferring two methyl groups from S-Adenosyl-L-methionine (SAM) to its substrate [28,29]. Therefore, in addition to finding SAM dependent methyl transferases, we also identified transcripts encoding URO-D CIMS domains.

### Combining KAAS-KEGG pathway analysis with domain searching for phenylpropanoid and probable downstream podophyllotoxin pathway genes

**Figure 4:**
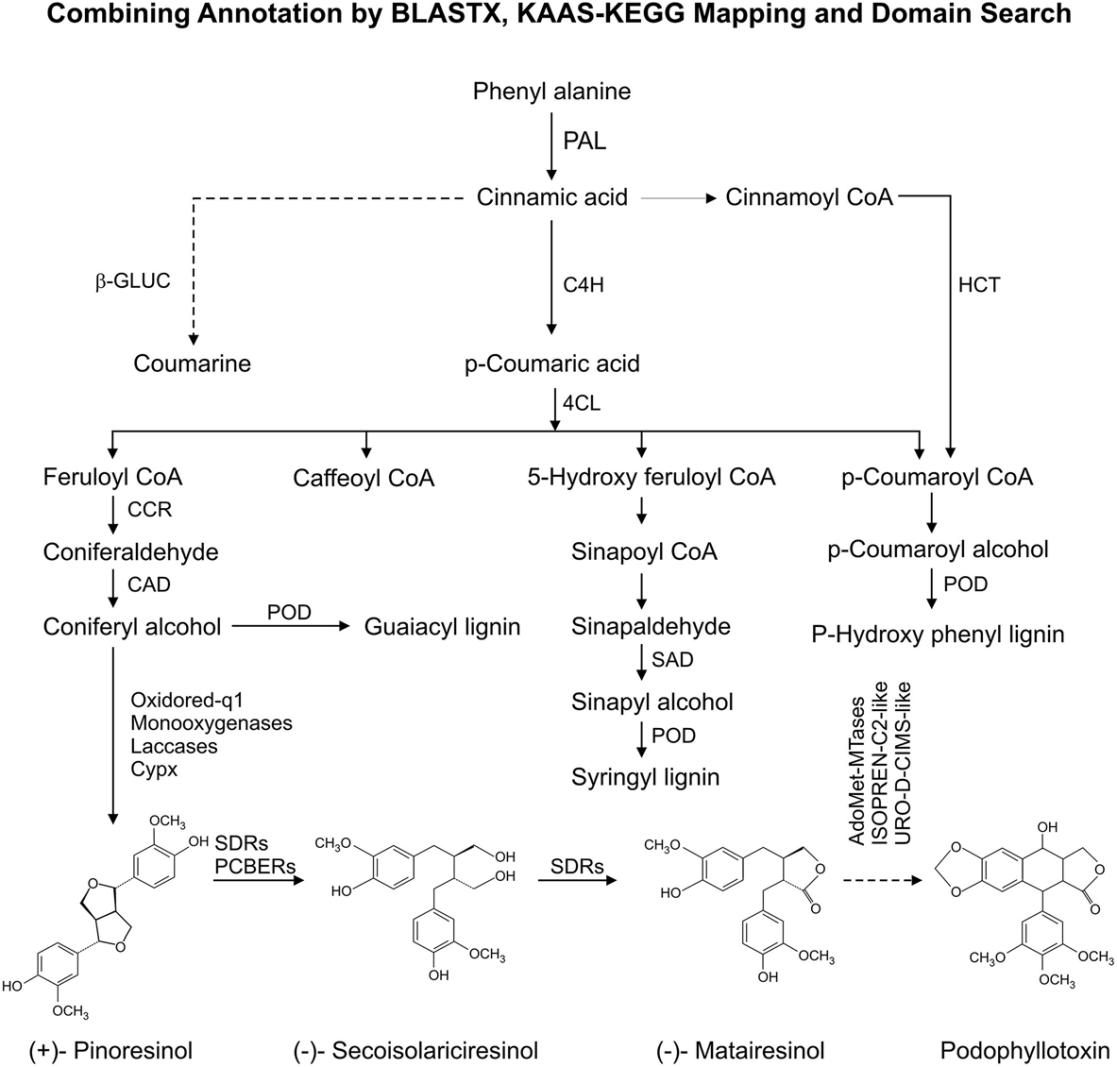
**Proposed scheme of for phenylpropanoids and podophyllotoxin biosynthesis in *P. hexandrum*.** A scheme combining annotation by BLASTX, KAAS-KEGG mapping and domain search for phenylpropanoid pathway transcripts and transcripts with domains that may be part of podophyllotoxin biosynthetic pathway from transcripts generated by Newbler default and optimized parameter assembly. PAL-Phenyl alanine ammonia lyase, β-GLUC, Beta glucosidase; C4H, cinnamate 4-hydroxylase; HCT, Hydroxy cinnamoyl transferase; 4CL, 4-coumarate ligase; CCR, cinnamoyl CoA reductase; CAD, Cinnamoyl alcohol dehydrogenase; POD, Peroxidase; SAD, Sinapyl alcohol dehydrogenase; SDR, Secoisolariciresinol dehydrogenase like; PCBER, phenylcoumaran benzylic ether reductase like; AdoMeT, MTase-Adenosyl Methionine dependent methyl transferase like; ISOPREN-C2-like, Isoprene-C2-like reductase; URO-D-CIMS-like, Uroporphyrinogen decarboxylase methyl transferase.

BLASTX analysis and KAAS-KEGG pathway mapping of transcripts from Newbler default and optimized parameter identified cDNAs encoding Phenylalanine ammonia lyase (PAL), hydroxyl cinnamoyl transferase (HCT), cinnamate-4-hydroxylase (C4H), 4-Coumarate Ligase (4CL), 4-Coumarate CoA Ligase (4CL), cinnamoyl reductase (CCR), CAD, sinapyl alcohol dehydrogenase (SAD), β-Glucosidase (β-GLUC) and POD as being involved in the phenylpropanoid pathway. Podophyllotoxin pathway is hypothesized to start with CAD converting coniferaldehyde to coniferyl alcohol, which then undergoes dirigent-mediated coupling to form pinoresinol. Specific reductases, dehydrogenases and methyl transferases are then believed to convert pinoresinol to podophyllotoxin. We surveyed the CDD results for cDNAs with domains that may represent genes of this pathway and identified transcripts containing Phenylcoumaran benzylic ether reductase (PCBER), SDGs, monooxygenases, SAM dependent methyl transferases and URO-D CIMS-like domains. A scheme has been presented combining the BLASTX annotation, KAAS-KEGG mapping and domain search for phenylpropanoid pathway transcripts and transcripts with domains that may be part of podophyllotoxin biosynthetic pathway ( Figure 4).

### Transcription factors related to secondary metabolism

**Figure 5:**
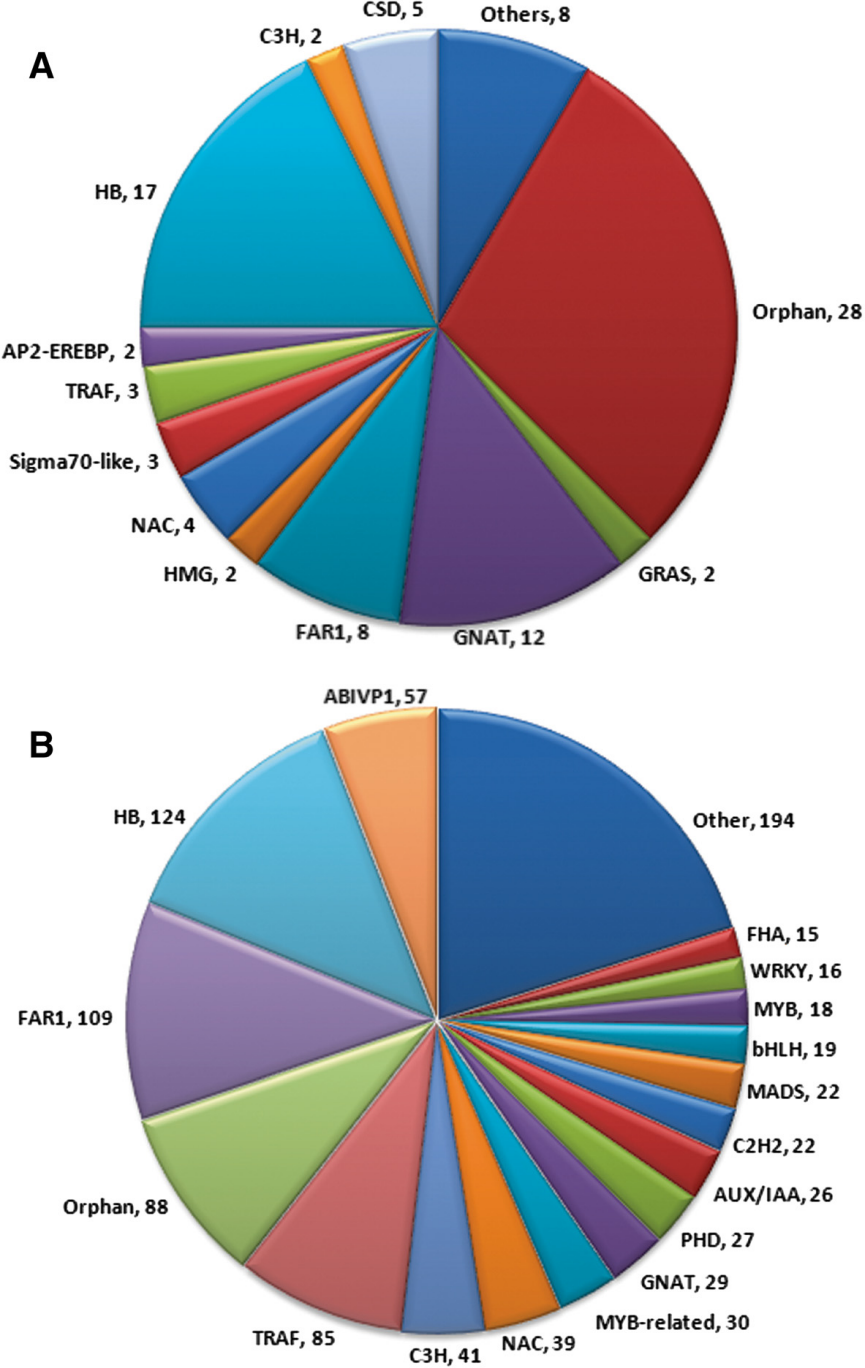
Transcription factors identified from transcripts generated by Newbler A) default and B) optimized parameter assembly.

Controlled transcription of biosynthetic genes is an important mechanism for regulating secondary metabolite production in plant cells [30]. Certain TFs are known to be involved in the regulation of secondary metabolism, such as R2R3-MYB, basic helix-loop-helix (bHLH) proteins like CrMYC2, AP2/ERF family proteins, WRKY, NAC, DOF, bZIP, HD-ZIP, and TFIIIA zinc finger TFs. We identified 96 transcripts from Newbler default assembly (Additional file 14) and 961 transcripts from optimized parameter assembly (Additional file 15) that may encode TFs (Figure 5A, B respectively). Amongst them, notable transcripts were AP2-EREBP, NAC, bHLH, MYB or MYB related, bZIP, mTERF, WRKY, C2C2-CO-like and C2C2-Dof. A number of plant MYB TFs regulating the phenylpropanoid biosynthetic pathway, identified from many species, including *Arabidopsis*, apple, grape, maize, petunia and snapdragon, most of which are R2R3-MYB TFs [31] can be correlated with our study as 48 transcripts coding for MYB or MYB related TFs have been identified from the optimized Newbler assembly. Again, R2R3-MYB transcription factor MYB12 in *A*. *thaliana* has been shown to function as a flavonol specific activator of flavonoid biosynthesis [32]. Transcriptional regulation of flavonoid biosynthesis, a major branch of phenylpropanoid pathway, controlled by a set of R2R3 MYB transcription factors, have been reported in several plants such as *Prunus persica*, *Epimedium sagittatum* as well [33,34]. Other than this TF, 18 transcripts coded for bHLH TFs have been identified here. The bHLH-domain of the maize R-gene is reported to participate in anthocyanin formation and serve as a link between flavonoid formation and histone modification [35]. Amongst the diverse functions, bHLH transcription factors also regulate the biosynthetic pathway of flavonoids, in several plant species [31]. 1 DOF family TF has been identified in our analyses. AtDOF4 is known to influence phenylpropanoid metabolism in an environmental and tissue-specific manner by positively regulating the production of hydroxycinnamic acids in the hypocotyl and flower buds, and negatively regulating flavonoid biosynthesis in pollen grains [36]. Together, TFs identified here and related to the phenylpropanoid pathway can be explored further in the regulation of podophyllotoxin biosynthesis.

### *In silico* SSR marker identification

SSRs can be divided into genomic SSRs and EST-SSRs. EST-SSRs are more evolutionary conserved than noncoding sequences and therefore have a relatively high transferability [33,37]. Next-generation sequencing has identified EST-SSRs in many plant species [38-41]. However, there have been no reports of EST-SSRs in *P. hexandrum* to date.

SSRs were identified with MISA search tool (http://pgrc.ipk-gatersleben.de/misa/), which is based on the criteria that a dinucleotide or a trinucleotide pattern should appear at least six times, and tetra, penta and hexa nucleotide patterns should appear five times each (Additional file 16 for Newbler default, Additional file 17 for Newbler optimized). SSR distribution and SSR mining of transcripts identified a total of 1,011 SSRs from 40,380 transcripts, with 94 transcripts containing more than one SSR. The most abundant repeat type was dinucleotides (68.6%) and the dominant tandem repeat motifs were (AT)6 and (AT)7 representing 19.4% and 25.7% respectively.

### Transcriptome wide survey of miRNA targets in *P. hexandrum* cell cultures

MiRNAs are known to regulate many developmental and effector genes at the posttranscriptional level [38,42]. Using oligonucleotide arrays, miRNAs have been shown to be differentially expressed between tissues and during the maturation of the fruit in the grapevine [43]. Wong *et al.*[44], predicted three wood related genes, flavonol synthase-like, xyloglucan fucosyltransferase and glucan synthase-like genes to be the targets of miR170, miR172 and miR319, respectively, and suggested that these miRNAs might be directly involved in regulation of the phenylpropanoid pathway and hemicellulose biosynthesis pathway. Downregulation of Flavonol synthase by miR170 would redirect the precursor 4-coumaroyl CoA to lignin biosynthesis.

We identified precursor and mature miRNAs in the *P. hexandrum* cell culture transcriptome, by searching the contigs and singlet in the public miRNA database (Additional file 18 and Additional file 19) [41,45]. Transcripts targeted by miRNAs that are possibly related to phenylpropanoid and podophyllotoxin biosynthesis include cytochrome b6, cytochrome p450 like, cell wall associated hydrolase, cell wall associated protein, laccase, and cytochrome f. Deoxypodophyllotoxin 6-hydroxylase is a cytochrome p450 dependent monooxygenase, that catalyzes the introduction of a hydroxyl group in position 6 of deoxypodophyllotoxin [46]. A cytochrome p450 protein is known to catalyze the biosynthesis of a lignan, (+)- sesamin, by forming two methylenedioxy bridges [47]. Laccases have auxiliary roles in stereoselective coupling to 8-8′ linked lignans [48].

### Comparative qRT-PCR of selected phenylpropanoid pathway genes in cell culture, callus and rhizome and podophyllotoxin accumulation in *P. hexandrum* cell culture

**Figure 6:**
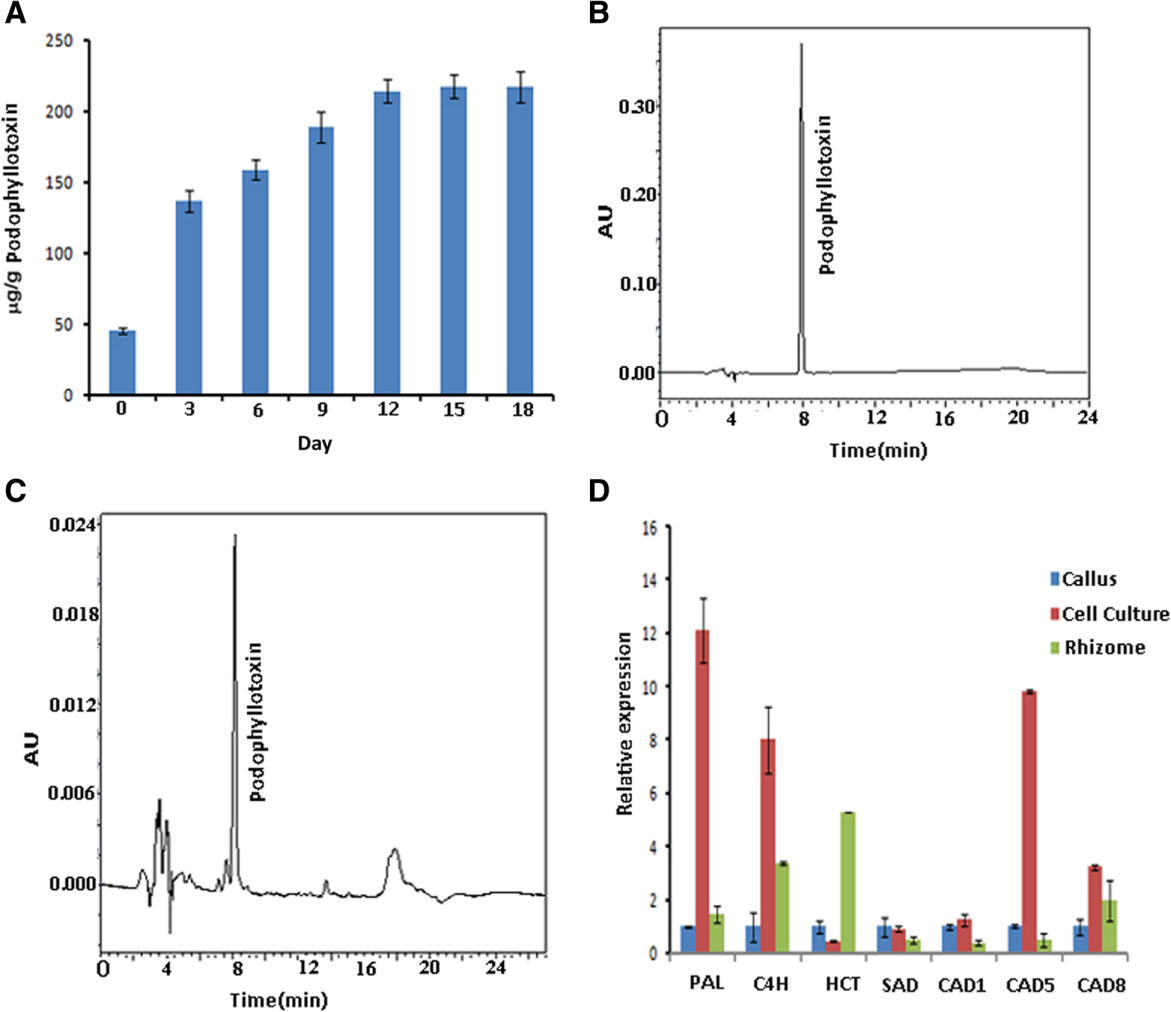
**Accumulation of podophyllotoxin and qRT-PCR of selected phenylpropanoids pathway genes. A)** Accumulation of podophyllotoxin in cell cultures from day1 to day 18 using HPLC. HPLC analyses for three biological replicates were done. **B)** Representative HPLC chromatogram of standard podophyllotoxin. **C)** Representative HPLC chromatogram of *P. hexandrum* cell culture extracts. **D)** qRT-PCR of selected phenylpropanoid pathway genes with reference to internal standard actin in callus, 12 day old cell suspension culture and rhizome of *P. hexandrum*.

Podophyllotoxin content in *P. hexandrum* rhizome is known to vary from 4% to 10% in reference to age, altitude, net photosynthetic rate, stomatal conductance, carbon uptake and number of leaves [49-52]. Green calli were found to have 40–50 μg/g podophyllotoxin which were used to generate cell suspension culture. Enhanced accumulation of podophyllotoxin was observed from 3 day old cell culture and increased up to 12 day (Figure 6A) as noted by HPLC analysis (Figure 6B, C). However, no significantly increased accumulation was observed till 18 days. Hence, we chose to compare relative gene expression profile of selected phenylpropanoid pathway transcripts amongst the calli, 12 day old cell culture, and the rhizome. Transcripts of CAD identified here, CAD1, CAD5, and CAD8 share sequence similarity to *Arabidopsis* CAD1, CAD5, and CAD8 respectively as identified by BLASTX analysis. Primers were designed from transcript contigs for qRT-PCR analysis (Table 3). *PAL* in 12 days cell culture shows 12.12 fold upregulation with respect to the callus, while upregulation of *PAL* expression levels in rhizome is insignificant (Figure 6D). *C4H* is upregulated in the cell culture and the rhizome by 8 and 3.4 folds respectively in comparison to that of callus. The high FPKM value of *C4H* in the 12 day cell culture samples may correlate with this observation (Table 3). Furthermore, upregulation of CAD 1,5, and 8 can be correlated as well with the FPKM value on the higher side.

**Table 3:** FPKM values of the transcripts used for qRT-PCR analysis

**Gene**	**Transcript No**	**FPKM**
*PAL*	transcript_1243	4.39
*C4H*	transcript_22196	205.32
*CAD8*	transcript_2809	13.57
*CAD1*	transcript_3581	10.61
*CAD5*	transcript_6353	3.30
*HCT*	transcript_28273	272.33
*SAD*	transcript_29195	118.10

## Conclusions

This report comprises a large, assembled and functionally annotated high throughput genomic resource for *P. hexandrum*. Our efforts to unravel the probable genes related to the podophyllotoxin biosynthetic pathway using the next-generation whole transcriptome sequencing of *P. hexandrum* identified almost all the known members of the phenylpropanoid pathway. The annotated transcripts represent a useful resource for subsequent isolation of podophyllotoxin pathway genes in *P. hexandrum*. In addition to pathway identification, the identification of EST-SSRs as molecular markers will be useful for conservation of *P. hexandrum*, which is an endangered species.

## Methods

### Sample preparation and 454 pyrosequencing

Calli were induced from mature leaves of *P. hexandrum* in MS medium [53] supplemented with 2.68 μM Napthyl acetic acid (NAA) and 8.88 μM Benzylaminopurine (BAP) [54]. Cell suspension cultures were initiated from freshly subcultured green calli of *P. hexandrum* in modified liquid MS medium [55] containing 60mM total N content, 1.25 mM potassium dihydrogen phosphate, 6% glucose and 11.41 μM Indole acetic acid (IAA). An inoculum of 5 g cells was used in 50 ml of cell suspension culture medium. Six flasks containing cell suspension cultures were shaken in the dark at 110 rpm for 12 days. The cells were collected by centrifugation at 1000 × g for 5 min, and immediately put in liquid nitrogen and used for RNA isolation. Total RNA was isolated from 12 days old cell suspension cultures using Purelink miRNA isolation kit (Invitrogen, CA, USA). Total RNA was quantified by NanoDrop technology, checked on a 1% denaturing agarose gel and on a bioanalyzer 2100 (Agilent Technologies, Palo Alto, CA). Removal of rRNA from total RNA was performed using RiboMinus Plant kit for RNA seq (Invitrogen cat. no. A10838-08) using the standard procedure and then concentrated by RiboMinus concentration module (cat no. K155005), according to the manufacturer’s instructions. Library preparation performed using a cDNA Rapid Library Preparation Method Manual-GS FLX Titanium Series, according to the manufacturer’s instructions. For transcriptome sequencing, 1 μg of Ribo-minus total RNA from each sample was used for fragmentation using ZnCl_2_ solution, followed by ds-cDNA synthesis using a standard cDNA synthesis kit (Roche). This ds-cDNA was then subjected to fragment end-repair followed by adaptor ligation using Rapid Library Prep kit (Roche). emPCR amplification of the cDNA library was performed according to the manufacturer’s instructions (Roche). Clonally amplified cDNA library beads obtained from the emPCR amplification reaction were deposited on a PTP for sequencing using pyrosequencing chemistry. The next-generation sequencing run for whole transcriptome analysis was performed on a Roche 454 GS FLX.

### *De novo* assembly

Raw reads obtained from 454 pyrosequencing were preprocessed by removing low quality reads, and adapter/ primer sequences using PRINSEQ. The high quality reads were uniqed and mapped to non-coding RNA database Rfam (v11.0) using gsMapper. Reads that mapped to ncRNAs sequences were excluded and remaining reads were used for further analysis.The preprocessed reads were then assembled using Newbler with default parameters and optimized parameters. Optimized parameters were set by checking “Use duplicate reads”, “Extend low depth overlaps”, “Reads limited to one contig” and “Single Ace file options”. The sequence data generated in this study have been deposited at NCBI in the Short Read Archive database under the accession (**SRX180870** and **SRX180386**).

### Functional annotation, GO mapping, pathway analysis, FPKM value determination and EST-SSR identification

Annotation of the transcripts was carried out using green plants of non-redundant (nr) protein database NCBI using BLASTX. GO mapping was carried out with BLAST 2GO (GO; http://www.geneontology.org). KEGG maps and an enzyme classification number (EC number) were built for pathway analysis. FPKM values for the transcripts were determined using the formula, FPKM = (Number of reads Mapped × 10^9^) / (Length of Transcript × Total Number of Reads). Here number of reads mapped were calculated by mapping reads on assembled transcripts using CLC Genomics Work bench with a mismatch, insertion, deletion cost of 2, 3 and 3 respectively. Potent EST-SSR markers were identified by MISA, a customized Perl script tool freely available for prediction of SSRs [56].

### Protein domains and transcription factor identification in *P. hexandrum*

Transcripts were searched against a conserved domain database (CDD v3.07) with an E-value cut-off of 0.01 for different domains. For the identification of transcription factor families represented in the *P. hexandrum* cell culture transcriptome, the transcript contigs were searched against all the transcription factor protein sequences at the plant transcription factor database (http://plntfdb.bio.uni-potsdam.de) using BLASTX with an E-value cutoff 1E-06.

### MiRNA target identification in *P. hexandrum* cell cultures

Conserved miRNAs and their target cDNAs, were found by aligning transcripts against the mature and precursor sequences of known plant miRNAs deposited in miRBase version 19 http://www.mirbase.org/ using CLC Genomic Work bench with a mismatch, insertion, deletion cost of 2, 3 and 3 respectively*.*

### Lignan extraction and high performance liquid chromatography (HPLC) analysis

Lignans were extracted from *P. hexandrum* cells [57]. In brief, 100 mg of cells were extracted with 2 ml ethanol for 20 min at 60°C in microtubes and sonicated for 15 min. The supernatant was collected after centrifugation and evaporated to dryness under a vacuum. Extracts were dissolved in methanol and used for HPLC analysis. Podophyllotoxin (Sigma-Aldrich, Bangalore, India) was used as a standard. Podophyllotoxin extractions were performed with three biological replicates.

For HPLC, a Waters 2998 photodiode-array detector was set at 290 nm, and separation was carried out using an XTerra RP18, 5 μm, (4.6 × 250 mm i.d.) column. Data analyses were performed with Empower 2 software. Chromatographic conditions were essentially as previously described [7] and standardized in our laboratory [29].

### Quantitative RT-PCR (qRT-PCR)

Total RNA was extracted with a Purelink miRNA isolation kit (Invitrogen) using three biological replicates. RNA (2 μg) was reverse transcribed with oligo(dT) primers using RevertAid H Minus First Strand cDNA Synthesis kit (Fermentas, USA). PCR amplification was performed with Power SYBR Green PCR Master Mix (Applied Biosystems, Japan) on an Applied Biosystems 7500 Real-Time PCR System (Applied Biosystems, USA). Relative expression levels were calculated using the ∆-∆Ct method. All primers for qRT-PCR of selected phenylpropanoid pathway genes have been designed from the sequences obtained from optimized Newbler assembly (Additional file 20). Actin primers were designed as reported (Acc. No. FL640971.1), Forward primer: 5′-CTCGGGAGGTGCCACCACC-3′ and Reverse primer 5′-GATGGAAGCTGCTGGGTATTCA-3′.

## Competing interests

The authors declare that they have no competing interests.

## Authors’ contributions

The study was conceived by SC and DB. SC collected fresh samples of *P. hexandrum* from CSIR-IHBT, Palampur, India. The plant material preparation and gene expression analyses were carried out by DB, RS and SH. RD is maintaining the un-treated and treated cell suspension cultures of *P. hexandrum*. SC and DB contributed to data analysis, bioinformatics analysis, and manuscript preparation. All authors had read and approved the final manuscript.

## Supplementary Material

Additional file 1 Distribution of transcript contigs according to length assembled by Newbler using default parameters (A) and optimized parameters (B). Click here for file

Additional file 2 Annotation by BLASTX assembles sequences generated using Newbler with default parameters. Click here for file

Additional file 3 Annotation by BLASTX assembles sequences generated using Newbler with optimized parameters. Click here for file

Additional file 4 E-value distribution, sequence similarity distribution, evidence code distribution for sequences, evidence code distribution for BLAST hits, annotation score distribution, annotation distribution, and GO-level distribution for transcripts generated by Newbler using default parameters. Click here for file

Additional file 5 E-value distribution, sequence similarity distribution, evidence code distribution for sequences, evidence code distribution for BLAST hits, annotation score distribution, annotation distribution, and GO-level distribution for transcripts by generated by Newbler using optimized parameters. Click here for file

Additional file 6 GO annotation of transcripts from Newbler default assembly. Click here for file

Additional file 7 GO annotation of transcripts from Newbler optimized assembly. Click here for file

Additional file 8 FPKM values of transcripts from Newbler default parameter assembly. Click here for file

Additional file 9 FPKM values of transcripts from Newbler optimized parameter assembly. Click here for file

Additional file 10 KEGG biochemical mapping of transcripts from Newbler default parameter assembly. Click here for file

Additional file 11 KEGG biochemical mapping of transcripts from Newbler optimized parameter assembly. Click here for file

Additional file 12 List of important domains that may be required for podophyllotoxin biosynthesis from sequences assembled using Newbler default parameters. Click here for file

Additional file 13 List of important domains that may be required for podophyllotoxin biosynthesis from sequences assembled using Newbler optimized parameters. Click here for file

Additional file 14 Complete list of transcription factors identified from transcripts generated by Newbler default parameter assembly. Click here for file

Additional file 15 Complete list of transcription factors identified from transcripts generated by Newbler optimized parameter assembly. Click here for file

Additional file 16 List of SSRs, SSR distribution and SSR mining of transcripts generated by Newbler default parameter assembly. Click here for file

Additional file 17 List of SSRs, SSR distribution and SSR mining of transcripts generated by Newbler optimized parameter assembly. Click here for file

Additional file 18 List of mature and precursor miRNAs with their respective transcript IDs for transcripts generated by Newbler default parameter assembly. Click here for file

Additional file 19 List of mature and precursor miRNAs with their respective transcript IDs for transcripts generated by Newbler optimized parameter assembly. Click here for file

Additional file 20 Primers for qRT-PCR of selected phenylpropanoid pathway genes. Click here for file
