# Supplementary figures and images for: De novo transcriptome analysis using 454 pyrosequencing of the Himalayan Mayapple, Podophyllum hexandrum
Source: BMC Genomics. 2013 Nov 1;14:748. doi: 10.1186/1471-2164-14-748 (PMC3840631; doi:10.1186/1471-2164-14-748)

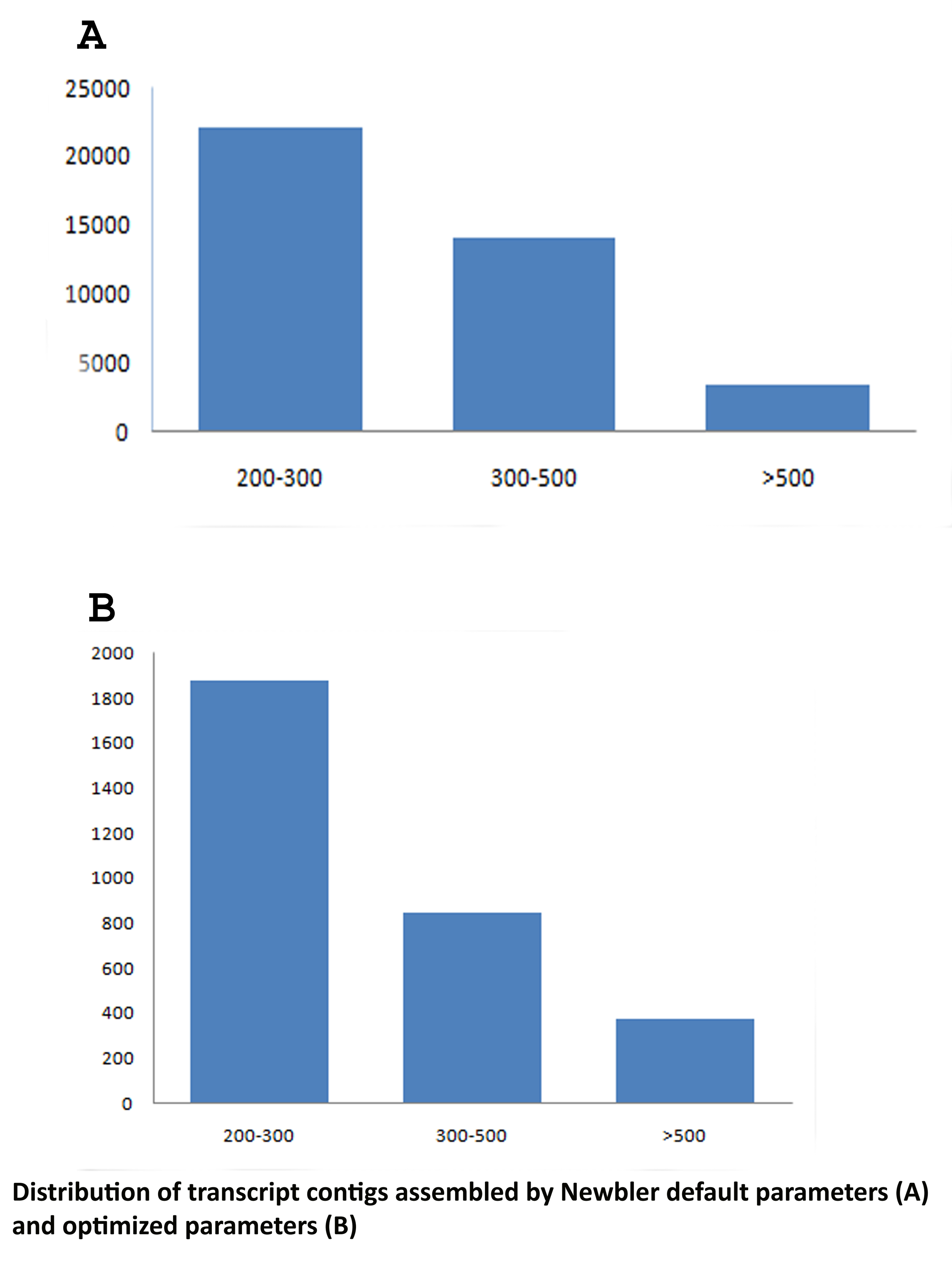

Supplement: Additional file 1 — Distribution of transcript contigs according to length assembled by Newbler using default parameters (A) and optimized parameters (B). [file 1471-2164-14-748-S1.jpg]

**A**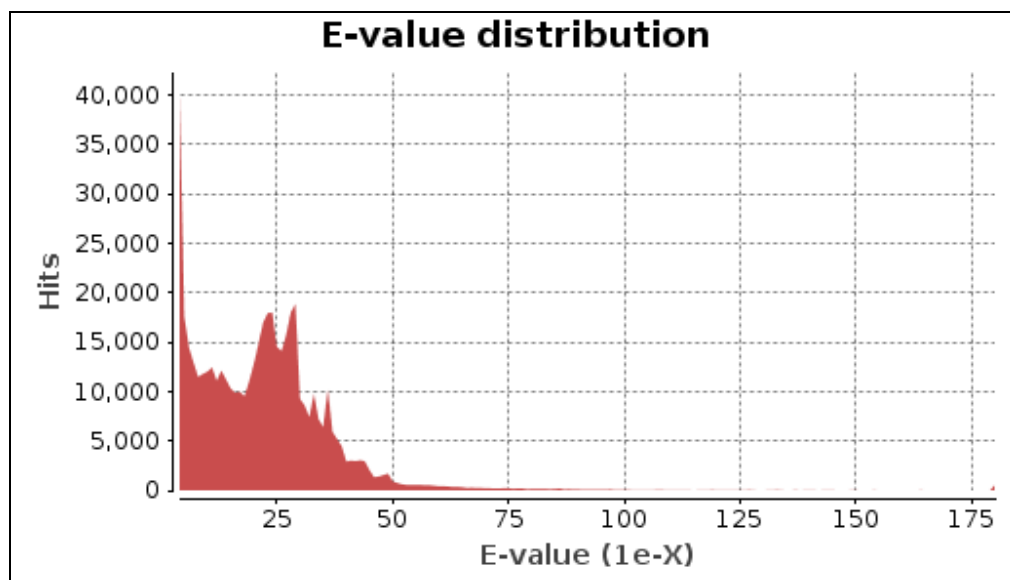**B**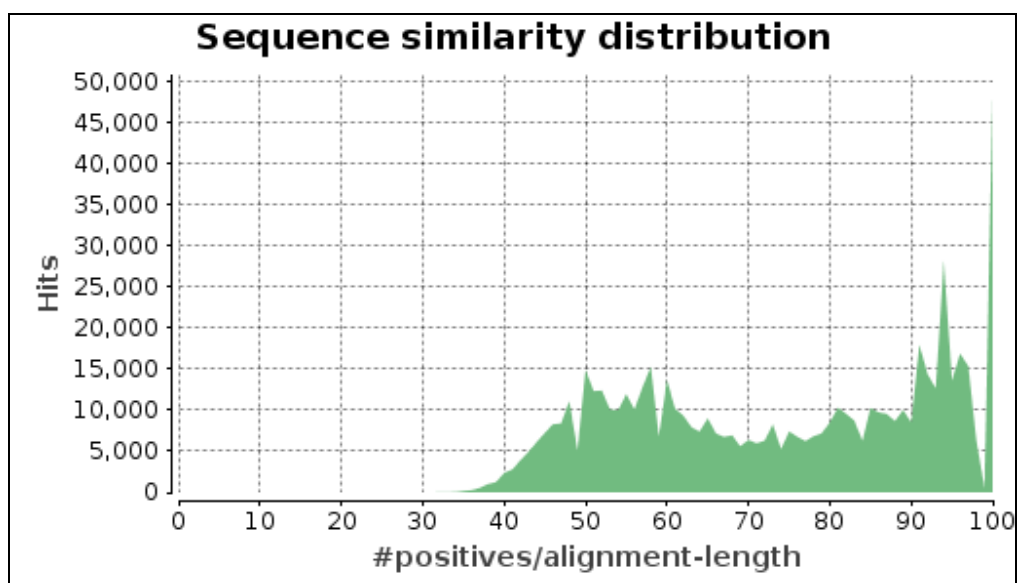

**C**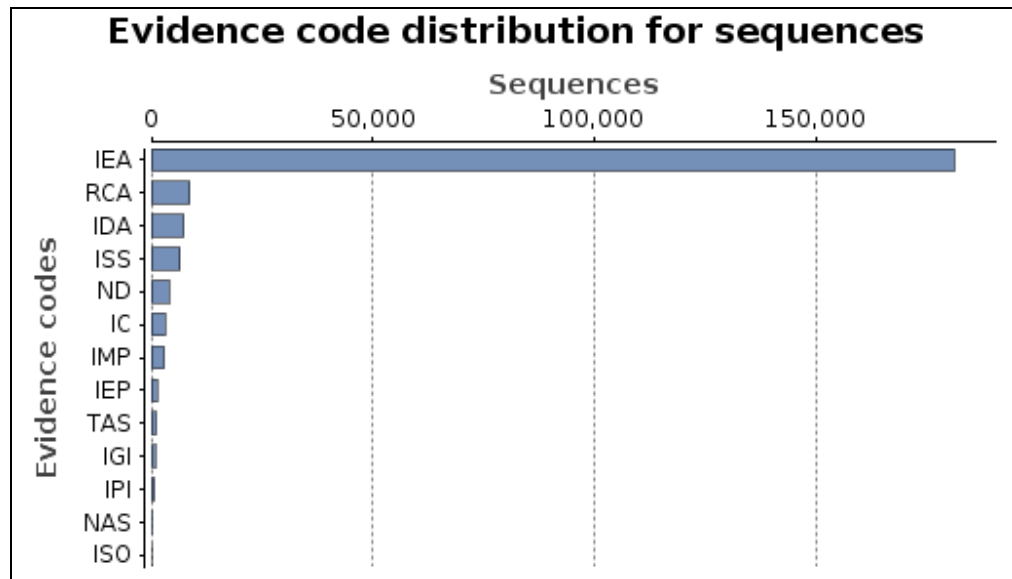**D**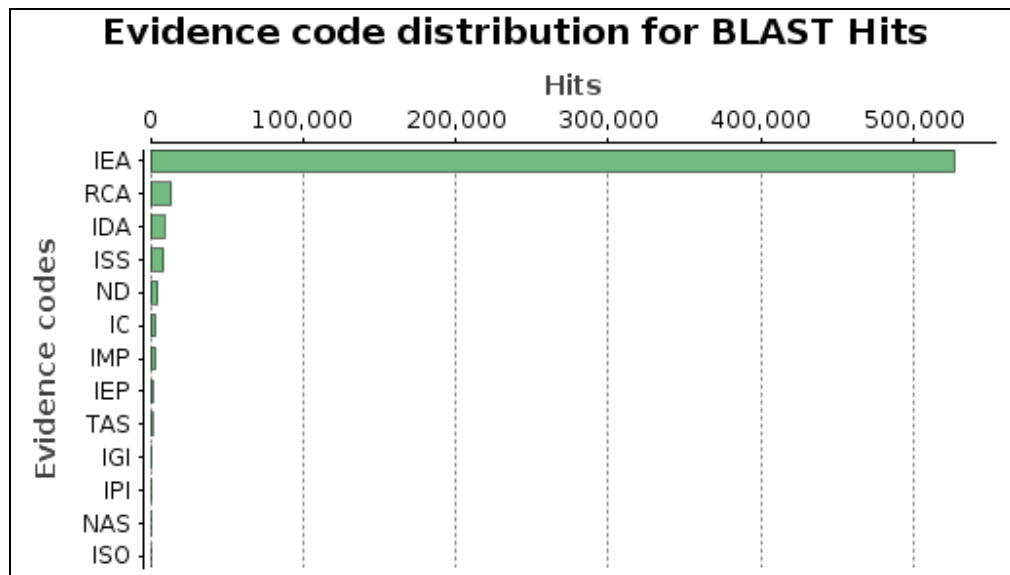

**E**

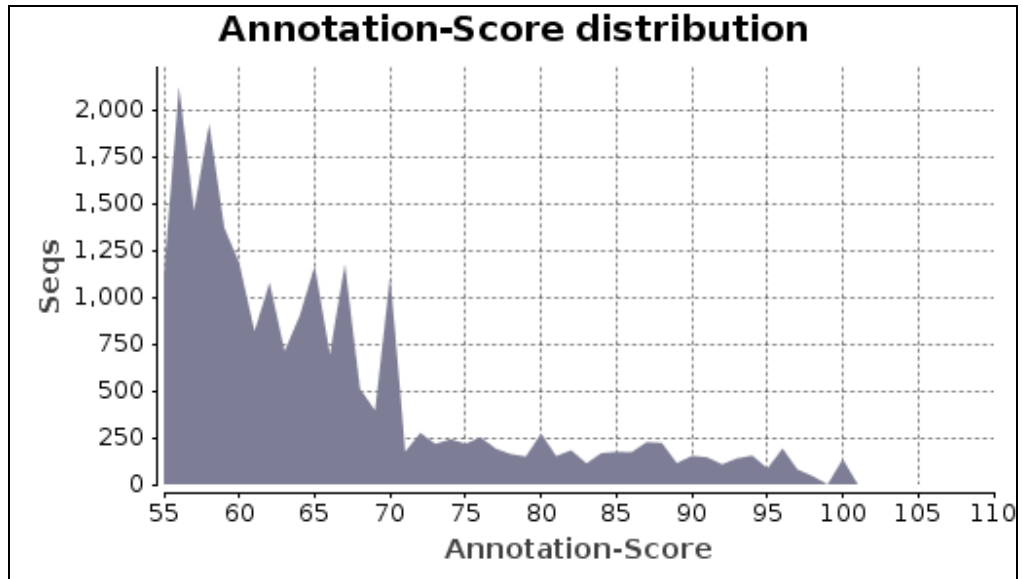

**F**

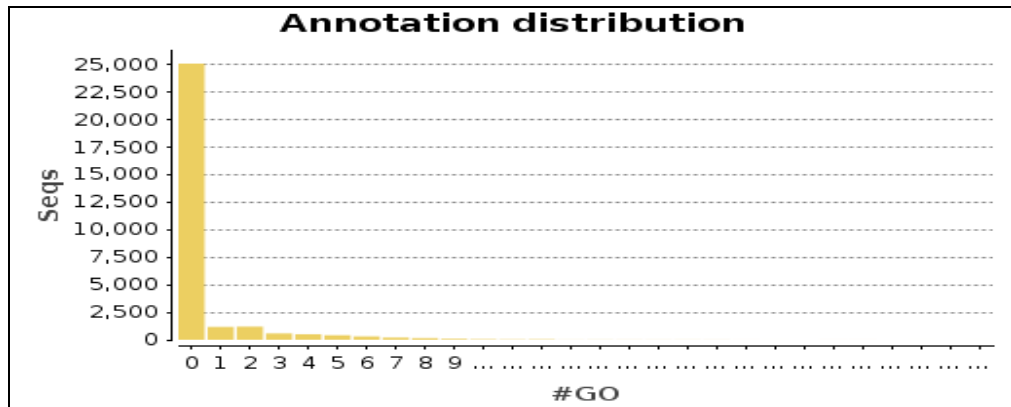

G

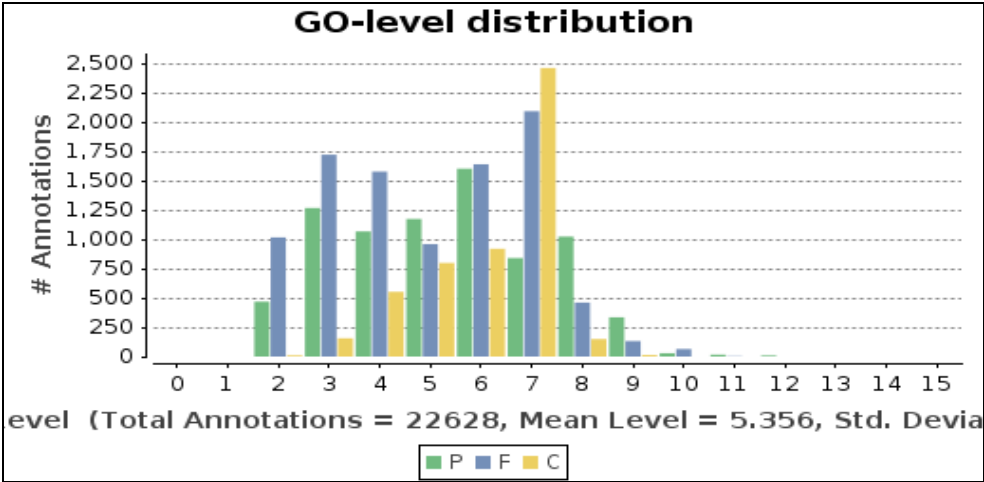

Supplement: Additional file 5 — E-value distribution, sequence similarity distribution, evidence code distribution for sequences, evidence code distribution for BLAST hits, annotation score distribution, annotation distribution, and GO-level distribution for transcripts by generated by Newbler using optimized parameters. [file 1471-2164-14-748-S5.pdf]
