# Supplementary material for: De novo transcriptome analysis using 454 pyrosequencing of the Himalayan Mayapple, Podophyllum hexandrum
Source: BMC Genomics. 2013 Nov 1;14:748. doi: 10.1186/1471-2164-14-748 (PMC3840631; doi:10.1186/1471-2164-14-748)
Supplement: Additional file 4 — E-value distribution, sequence similarity distribution, evidence code distribution for sequences, evidence code distribution for BLAST hits, annotation score distribution, annotation distribution, and GO-level distribution for transcripts generated by Newbler using default parameters. [file 1471-2164-14-748-S4.pdf]

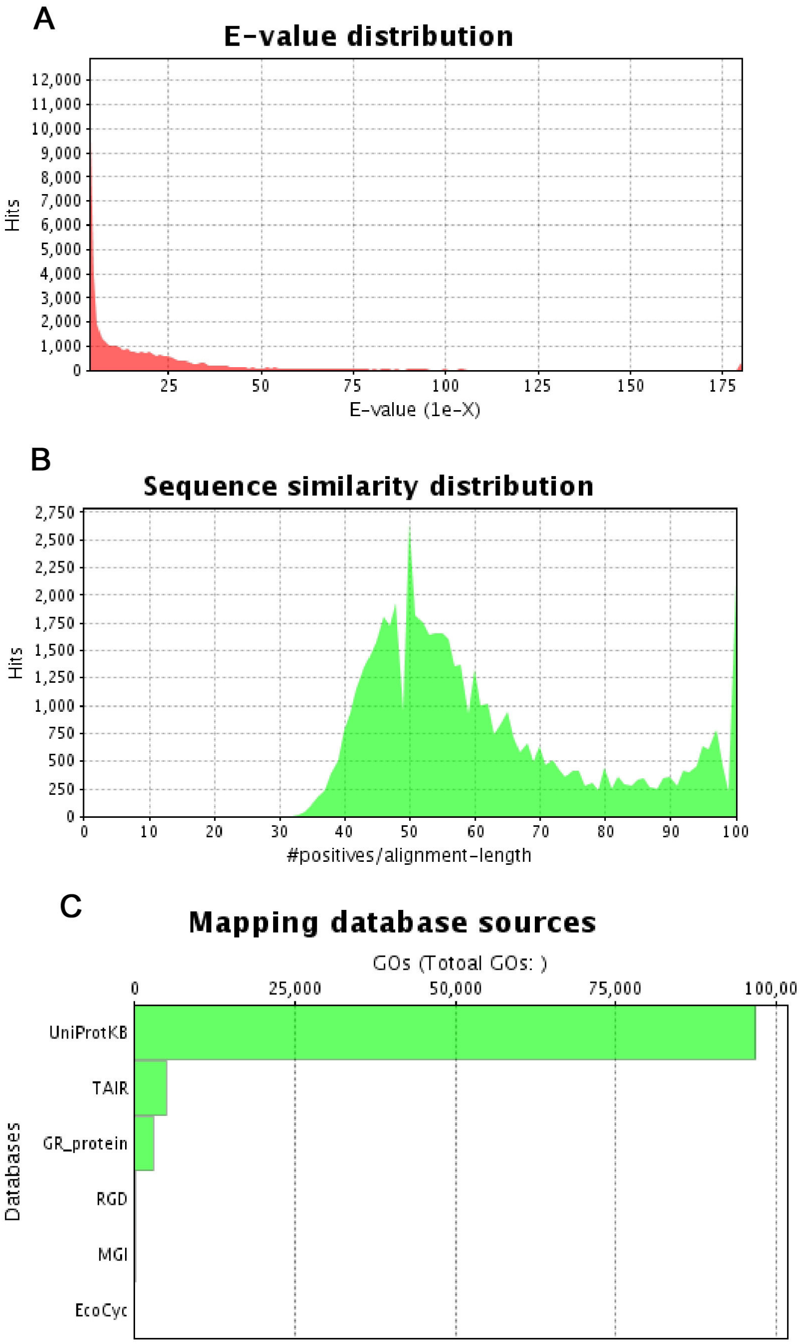
A
E-value distribution
Hits
E-value (1e-X)
B
Sequence similarity distribution
Hits
#positives/alignment-length
C
Mapping database sources
GOs (Totoal GOs:)
Databases
UniProtKB
TAIR
GR_protein
RGD
MGI
EcoCyc

D

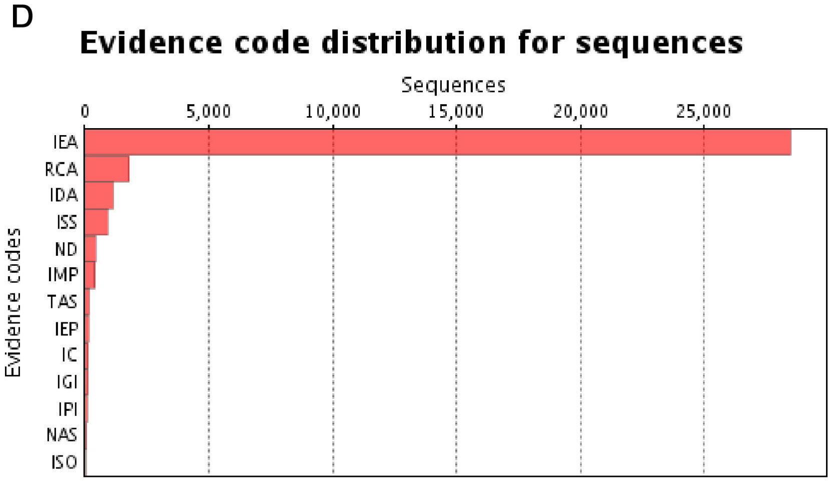
Evidence code distribution for sequences
Sequences
0
5,000
10,000
15,000
20,000
25,000
Evidence codes
IEA
RCA
IDA
ISS
ND
IMP
TAS
IEP
IC
IGI
IPI
NAS
ISO

E

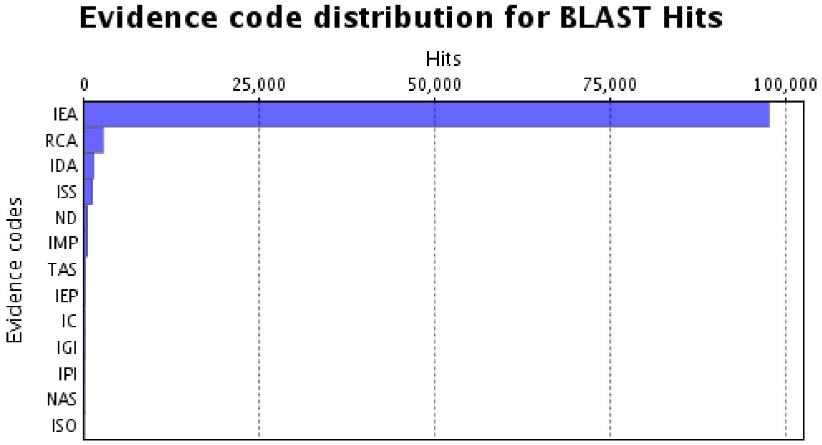
Evidence code distribution for BLAST Hits
Hits
0
25,000
50,000
75,000
100,000
Evidence codes
IEA
RCA
IDA
ISS
ND
IMP
TAS
IEP
IC
IGI
IPI
NAS
ISO

F

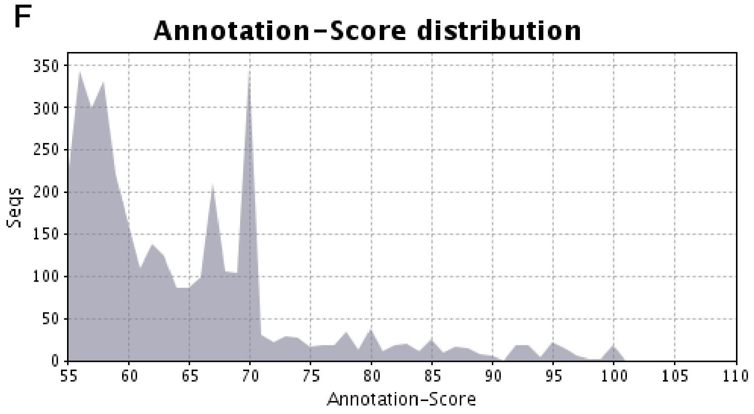
Annotation-Score distribution
Seqs
350
300
250
200
150
100
50
0
55
60
65
70
75
80
85
90
95
100
105
110
Annotation-Score

G

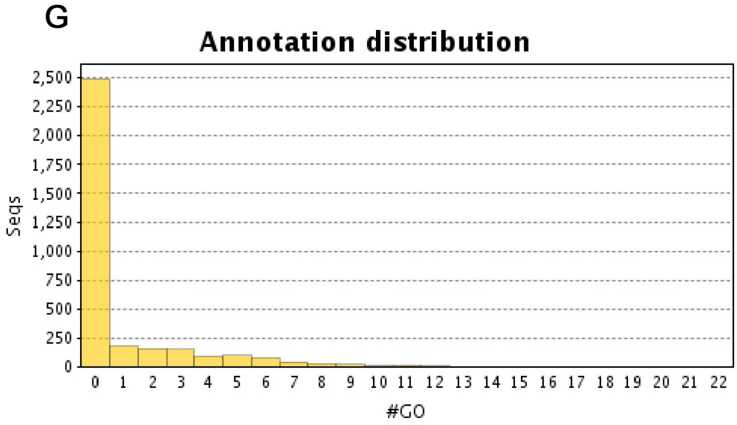
Annotation distribution
2,500
2,250
2,000
1,750
1,500
1,250
1,000
750
500
250
0
Seqs
0 1 2 3 4 5 6 7 8 9 10 11 12 13 14 15 16 17 18 19 20 21 22
#GO

H

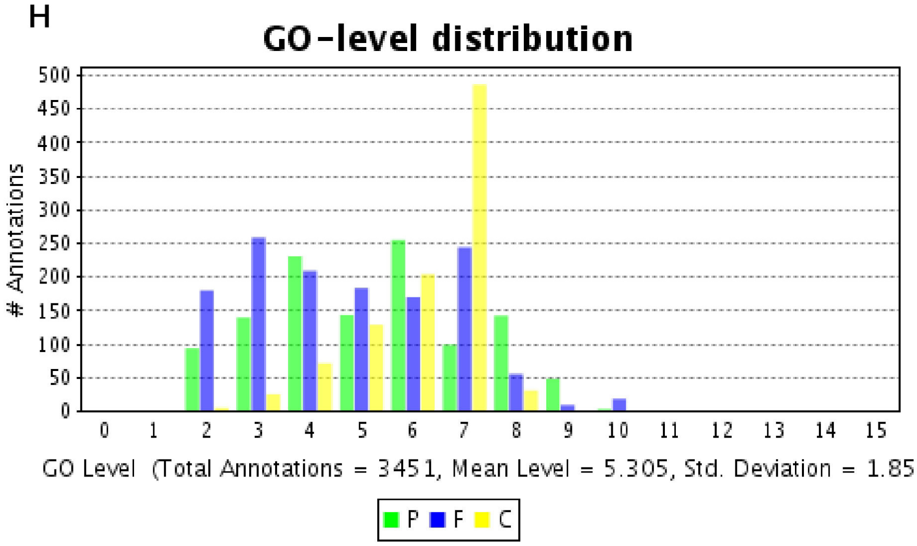
GO-level distribution
500
450
400
350
300
250
200
150
100
50
0
Annotations
0 1 2 3 4 5 6 7 8 9 10 11 12 13 14 15
GO Level (Total Annotations = 3451, Mean Level = 5.305, Std. Deviation = 1.85
P F C
