# Supplementary material for: De novo transcriptome analysis using 454 pyrosequencing of the Himalayan Mayapple, Podophyllum hexandrum
Source: BMC Genomics. 2013 Nov 1;14:748. doi: 10.1186/1471-2164-14-748 (PMC3840631; doi:10.1186/1471-2164-14-748)
Supplement: Additional file 20 — Primers for qRT-PCR of selected phenylpropanoid pathway genes. [file 1471-2164-14-748-S20.doc]

Additional File 20: Primer sequences used in Quantitative RT-PCR

| Primer | Sequence (5’-3’) |
| --- | --- |
| PAL Forward Primer | 5’-TCCACCATCGGCACCAGACG-3’ |
| PAL Reverse Primer | 5’-TGCGAATCGCATCGGCTGGG-3’ |
| C4H Forward Primer | 5’-GAGGGCCGCCATGAATTACAAT-3’ |
| C4H Reverse Primer | 5’-ACGATCCATCCCAACTAAGAACG-3’ |
| CAD8 Forward Primer | 5’-GATGGGCTGCAAGAGATTCATC-3’ |
| CAD 8 Reverse Primer | 5’-TAATTCTCTAGGTGGTGGACACA-3’ |
| CAD1 Forward Primer | 5’-GATAACCTCAATGTCAGCTGTTAT-3’ |
| CAD 1Reverse Primer | 5’-GCACAATGGATGGTATCATCGAC-3’ |
| CAD5 Forward Primer | 5’-CTTTGCGCCCTATCCTATTTGC-3’ |
| CAD5 Reverse Primer | 5’-TGCGCTGGCGCAGCTGCAG-3’ |
| HCT Forward Primer | 5’-AACACATGCAAGTCGAACGGGC-3’ |
| HCT Reverse Primer | 5’-GTCGTCGCCTTGGATGTAGCC -3’ |
| SAD Forward Primer | 5’- CGACTCGGGGCGTGGACCG -3’ |
| SAD Reverse Primer | 5’-TCCGGACTCCCTAACGTTTCG -3’ |
